# *Eucamaragnathus desenderi*, a new ground beetle species from Africa (Coleoptera, Carabidae)

**DOI:** 10.3897/zookeys.100.1521

**Published:** 2011-05-20

**Authors:** Thorsten Assmann, Claudia Drees, Andrea Matern, Andreas Schuldt

**Affiliations:** 1 Institute of Ecology and Environmental Chemistry, Leuphana University Lüneburg, Scharnhorststr. 1, D-21332 Lüneburg, Germany; 2 Tel Aviv University, George S. Wise Faculty of Life Sciences, Department of Zoology, The National Collections of Natural History, Tel Aviv 69978, Israel

**Keywords:** Coleoptera, Carabidae, Hiletini, new species, Zambia, South Africa, identification key

## Abstract

*Eucamaragnathus desenderi* **sp. n.**, a new ground beetle species of the tribe Hiletini, is described from eastern and southern Africa and dedicated to the recently deceased Belgian carabidologist Konjev Desender. The new taxon is known so far from localities in Zambia (Mukuku, southeast of Mansa) and in South Africa (Bothaville, south of Klerksdorp). The new species belongs to the *Eucamaragnathus castelnaui* group and is characterized by shape of pronotum, smooth or sparsely punctate pronotal transverse impression, characters of male genitalia and elytral striae continued to the apex. Illustrations of the habitus, the median lobe and its internal sac and several other morphological features are presented. An updated identification key to the African *Eucamaragnathus* species is given.

## Introduction

The pantropically distributed ground beetle tribe Hiletini is only poorly known, mainly because its members are rarely represented in collections. Erwin & Stork (1985) describe in their revision 20 species arrayed in two genera. Since that time no further species have been described.

Consequently we were surprised to find a series of an *Eucamaragnathus* species among other ground beetles caught by the Czech coleopterologists Miroslav Snižek and Vladimír Tichý in southern and eastern Africa. The examination of the material revealed that the specimens belong to a new species. Here we describe the species and dedicate it to our deceased colleague and friend Konjev Desender due to his exceptional engagement in the fields of ground beetle ecology, evolutionary biology and taxonomy.

## Material

The material examined is housed in the collections listed below:

CAM	Collection of the Africa Museum, Tervuren, Belgium

CAS	Working collection Th. Assmann, Bleckede, Germany (type material will be given to Zoologische Staatssammlung München)

CFA	Working collection Sergio Faccini, Modena, Italy

CMA	Working collection Werner Marggi, Thun, Switzerland

CSH	Working collection P. Schnitter, Halle, Germany

CSS	Working collection P. Schüle, Stuttgart, Germany

CST	Working collection W. Starke, Warendorf, Germany (type material will be given to Westphalian Museum of Natural History, Münster, Germany)

CWR	Working collection D.W. Wrase, Berlin, Germany

## Methods

Measurements were made at a magnification between 12.5× and 50×, using an ocular micrometer in a Leica MZ 95 stereobinocular microscope. The following measurements are used in the description: Total body length is measured from the tip of the mandibles to the apex of the right elytron as the maximum linear distance; the width of the head (HW) as the maximum linear distance across the head, including the compound eyes; the length of the pronotum (PL) from the anterior to the posterior margin along the midline; the length of the elytra (EL) from the basal margin to the apex of the right elytron as the maximum linear distance; the maximum width of the pronotum (PW) and elytra (EW) at their broadest point; the width of the pronotal base (PBW) between the tip of the posterior angles; the width of the pronotal apex (PAW) between the tip of anterior angles.

### Microsculpture was examined at a magnification of 100×

Dissections were made using standard techniques; genitalia were preserved in a mixture of polyvinylpyrrolidon, sorbitol and glycerol on acetate labels (Lompe 1989), and pinned beneath the specimens from which they had been removed. The photographs were taken with an Olympus E-330 digital camera in combination with a Leitz MZ 95. Post-processing was done in Adobe Acrobat Professional 7.0. To achieve sufficient depth of focus, up to 20 planes were captured which were copied to separate layers, and the out-of-focus planes were masked by a stacking programme (Combine Z5).

## Description

### Eucamaragnathus desenderi

Assmann, Drees, Matern & Schuldt sp. n.

urn:lsid:zoobank.org:act:7F180D34-C5F8-4D79-AC05-E2F727CAD2E3

http://species-id.net/wiki/Eucamaragnathus_desenderi

#### Type material:

Holotype male: „ZAMBIA NE. 2004 / 240 km SE Mansa / 25 km SE Mukuku / 29.11. Snižek, Tichý” (CAS). Paratypes: 13 males and 8 females, same as holotype (CAS, CFA, CST, CSH, CSS, CWR). 2 males and 4 females: „RSA, NW prov. 2001 / Klerksdorp, 20 km W / of Bothaville, Vaal riv. / M. Snižek lgt. 12.1.“ (CAS, CWR).

#### Diagnosis:

**Figure 1.**
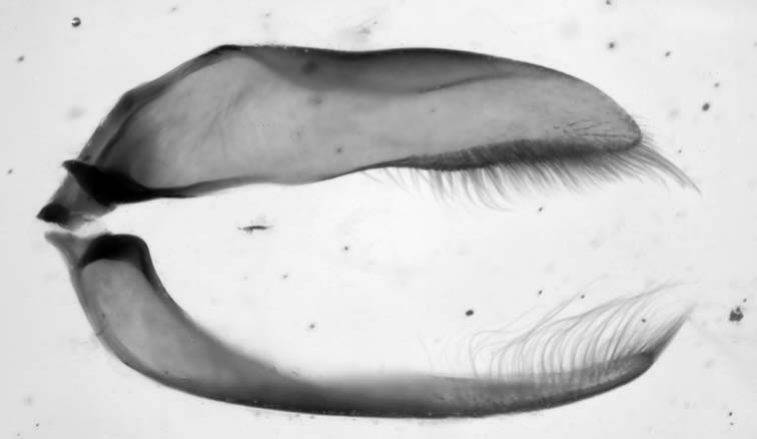
*Eucamaragnathus desenderi* sp. n., habitus; holotype.

A macropterous species of average size for the *Eucamaragnathus castelnaui* group, black, pronotum transverse, sides sinuate with posterior angles acute, transverse anterior impression punctulate, transverse posterior impression strongly punctate, elytral striae continued to apex. Habitus see Fig. 1.

#### Description:

Body length 8.8 – 10.6 mm; width 3.6 – 4.0 mm (holotype 10 mm and 3.8 mm, respectively).

##### Colour:

Black, without iridescence, not metallic; mandibels, mouth-parts, antennae, and tarsi partly infuscate.

##### Head

**Figure 2.**
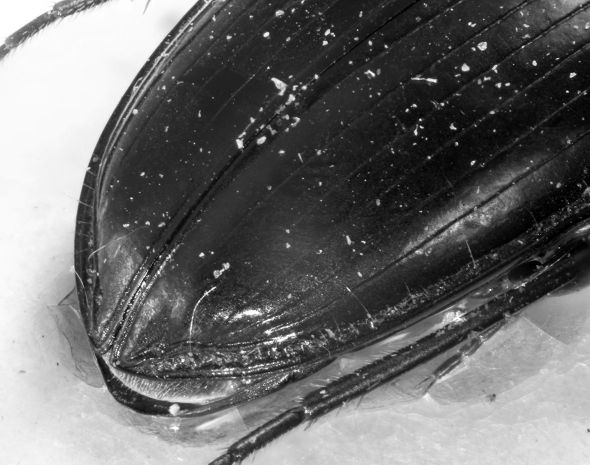
*Eucamaragnathus desenderi* sp. n., basal part of head, pronotum, basal part of elytra; holotype.

(Figs 1 and 2) large, about one fourth less wide than pronotum (HW: 2.0 – 2.4 mm, holotype: 2.3 mm; ratio HW/PW: 0.75 – 0.78). Eyes fairly large, their diameter (seen in dorsal view) about four tenth of head width; protected posteriorly by lateral extension of the cranium. Antennae robust, scape longer than the following 4 antennomeres, antennomeres 5 – 11 with dense and fairly fine setae. Mesal edge of mandibles markedly serrate (mandible teeth triangular shaped). Two pairs of supraorbital furrows. Frons not punctate, except basal close to pronotal anterior margin.

##### Pronotum

(Fig. 2) transverse (PW: 2.6 – 3.1 mm, holotype: 3.0 mm; PL: 1.9 – 2.2, holotype: 2.0 mm), widest prior to middle (basally of lateral seta). Pronotum at the base broader than at the apex (PAW: 2.2 – 2.6 mm, holotype: 2.5 mm; PBW: 2.3 – 2.8 mm, holotype: 2.7 mm). Anterior margin moderately straight; anterior angles pronounced, but rounded; lateral sides clearly sinuate; posterior angles acute, basal margin curved. Anterior transverse impression sparsely punctulate; lateral beads deep, not punctate; basal transverse impression deep, markedly punctate and connecting basal foveae; basal foveae deep, punctate and delimited externally by a keel-like carina without punctations.

##### Legs

(Fig. 1) similar to those found in other Hiletini species. Males with small tooth on profemur. Single long guard seta of tarsus 5 much longer than claws. Males with spatulate adhesive setae beneath protarsi 1 – 3 and mesotarsus 1.

##### Elytra

**Figure 3.**
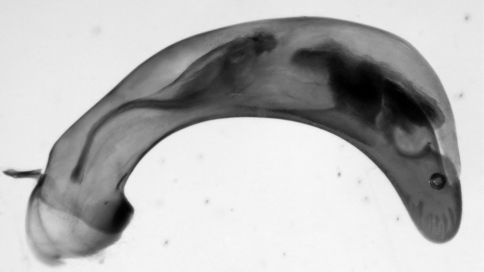
*Eucamaragnathus desenderi* sp. n., apex of elytra; paratype.

(Figs 1 and 3) with pronounced humeri, slightly enlarged to the end of the second third (EL: 4.8 – 5.9 mm, holotype: 5.75 mm; EW: 3.3 – 3.9 mm, holotype: 3.7 mm). Basal margin reduced, reaching 6th interval. Scutellar striae short; elytral striae deep and punctate, at the apex less impressed, but well visible; intervals flat, at the apex slightly convex. Discal setae of third stria in punctiform depressions.

##### Surface

with microsculpture of irregular and weak mesh patterns, meshes mainly transverse; a clear micropunctation on head, pronotum and elytra (20× magnification); surface shiny.

##### Male genitalia

**Figure 4.**
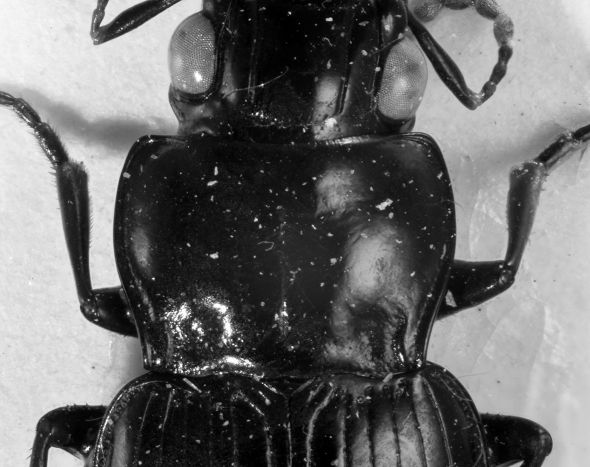
*Eucamaragnathus desenderi* sp. n., male genitalia, left lateral aspect of median lobe (aedeagus); paratype.

**Figure 5.**
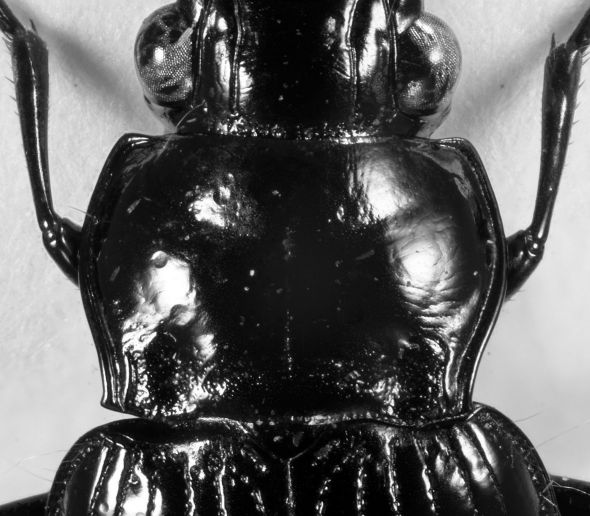
*Eucamaragnathus desenderi* sp. n., male genitalia, ventral aspect of parameres; paratype.

(Figs 4 and 5). Median lobe with ostium dextral. Both parameres multisetiferous, the setae of the narrow right paramere are longer than those of the broad left one.

#### Comparisons:

Due to form of mandible teeth and long single guard seta of last tarsomere the new species belongs to the genus *Eucamaragnathus* Jeannel, 1937. The small tooth of profemora in males, the dextral position of the ostium of the aedeagus and elytral striae continued to the apex place the new species in the *Eucamaragnathus castelnaui* (Bocandé, 1849) group (cf. Erwin and Stork 1985) which is exclusively distributed in Africa.

**Figure 6.**
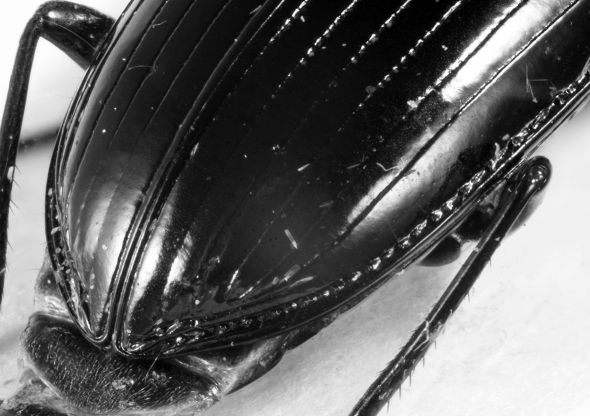
*Eucamaragnathus fissipennis*, basal part of elytra.

**Figure 7.**
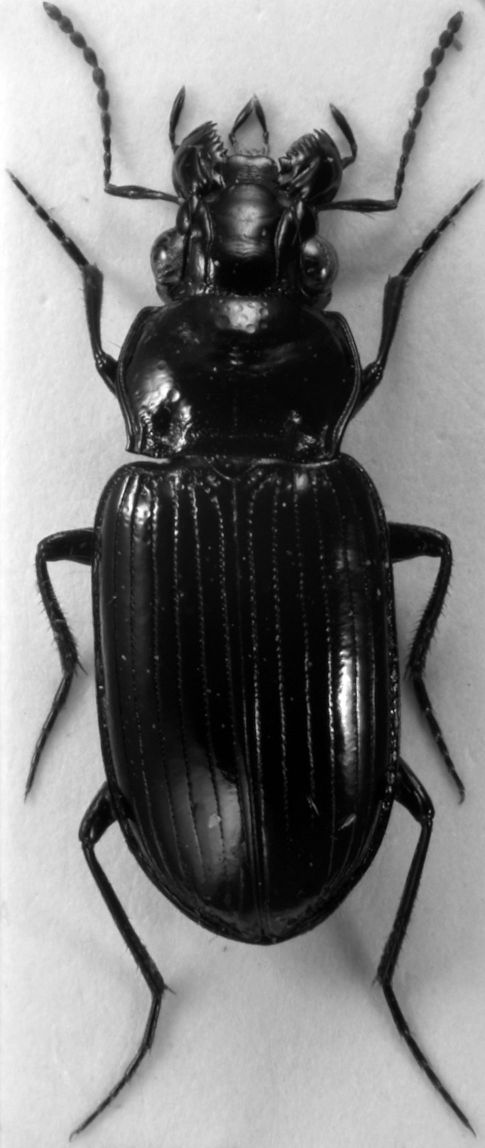
*Eucamaragnathus fissipennis*, apex of elytra.

The new species is similar to *Eucamaragnathus castelnaui* and *Eucamaragnathus fissipennis* (Ancey, 1882). The best character to separate *Eucamaragnathus desenderi* sp. n. from the nominate species of the group is the shape of the pronotum and especially the weak punctation of the pronotal anterior impression which is markedly punctate in *Eucamaragnathus castelnaui*. In comparison to the other species of the group, *Eucamaragnathus desenderi* sp. n. has acute pronotal anterior angles, but they are less produced than in *Eucamaragnathus oxygonus* Chaudoir, 1861. Moreover the median lobe, especially its internal sac structures, of *Eucamaragnathus desenderi* sp. n. differs from all other species of the given group. From *Eucamaragnathus fissipennis* the new species can be easily distinguished by stronger punctation of posterior transverse impressions of pronotum (Figs 2 and 6), stronger punctation of elytral striae, which are weaker at the apex, but still well visible (Figs 3 and 7) and a microsculpture with stronger punctation.

From *Eucamaragnathus bocandei* (Alluaud, 1914), which forms an own species group, the new species differs by its strong punctation of pronotal posterior impression and from *Eucamaragnathus suberbiei* (Alluaud, 1914) it can be separated by the size of tooth on ventral surface of profemur in males.

For better distinction we present an identification key for the known members of the African *Eucamaragnathus* species (see below).

#### Etymology:

It gives us great pleasure to dedicate this species to the memory of Konjev Desender, the well known Belgian carabidologist who recently deceased. We had many scientific meetings, excursions and productive collaborations with him, and we will honor his memory. An obituary is given by Lövei (2011) including a list of his publications.

#### Distribution:

Up to now *Eucamaragnathus desenderi* sp. n. is only known from the two sites in Zambia and South Africa. The population from Zambia (close to the border to Congo) lies in the tropical part of Africa fitting well to the main distribution area of the tribe in tropical Africa. In contrast, Bothaville in South Africa, the other site from where *Eucamaragnathus desenderi* sp. n. is known, is located between the 27th and 28th degrees of southern latitude, doubtless in the subtropical realm, and seems to be the most southern known record of a Hiletini species in Africa (and worldwide). The wide distribution of *Eucamaragnathus desenderi* sp. n. in Africa is not unusual for a Hiletini species (cf. the large distribution areas of *Hiletus alluaudi* (Jeannel, 1937) and *Eucamaragnathus fissipennis*, Erwin and Stork 1985).

*Eucamaragnathus desenderi* sp. n. seems to co-occur with *Eucamaragnathus fissipennis* which is distributed in tropical East Africa and south-eastern Africa. *Eucamaragnathus oxygonus* is known only from one locality in South Africa. All other African species of the genus *Eucamaragnathus* show – so far known – an allopatric distribution (*Eucamaragnathus suberbiei* is an endemic of Madagascar, *Eucamaragnathus castelnaui* and *Eucamaragnathus bocandei* occur exclusively in tropical western Africa, Erwin and Stork 1985).

#### Habitat:

The specimens were caught at light and habitat preferences are therefore unknown. Together with the holotype of *Eucamaragnathus desenderi* sp. n., a single Hiletini specimen of *Hiletus katanganus* Basilewsky, 1948 has been found. We compared this specimen of the rarely recorded species with the type material preserved in the Africa Museum (collection of Basilewsky) and detected morphological differences. Without more material (especially males) it seems to be impossible to assign specimens conclusively to this species (see also the note in Erwin and Stork 1985: 431).

#### Key to the African species of *Eucamaragnathus* Jeannel

This new identification key is based on the one presented by Erwin & Stork (1985), but it is modified and illustrated additionally.

**Table:** 

1.	Elytral stria 2 not continued to the apex, ending just behind last discal seta (Fig. 7)	*Eucamaragnathus fissipennis* (Ancey, 1882)
–	Elytral striae 1 – 4 continued to apex (Fig. 3)	2
2.	Pronotum with basal impression rugosely punctate. From continental Africa	3
–	Pronotum with basal impression smooth, no traces of punctation. From Madagascar	*Eucamaragnathus suberbiei* (Alluaud, 1914)
3.	Pronotum with anterior angles markedly produced, sides barely sinuate behind (Fig. 3h in Erwin & Stork 1985)	*Eucamaragnathus oxygonus* (Chaudoir, 1861)
–	Pronotum sides sinuate, more or less cordiform	4
4.	Male with tubercle on sternum VI	*Eucamaragnathus bocandei* (Alluaud, 1914)
–	Male without any specific character on sternum VI (except 1 pair of setae)	5
5.	Pronotum with anterior transverse impression markedly punctate, punctation similar to that of the posterior transverse impression of pronotum	*Eucamaragnathus castelnaui* (Bocandé, 1849)
–	Pronotum with anterior transverse impression with only few punctures, punctuation less strong than on the posterior transverse impression of pronotum	*Eucamaragnathus desenderi* sp. n.

## Supplementary Material

XML Treatment for Eucamaragnathus desenderi
